# Association of Polyphenol Biomarkers with Cardiovascular Disease and Mortality Risk: A Systematic Review and Meta-Analysis of Observational Studies

**DOI:** 10.3390/nu9040415

**Published:** 2017-04-22

**Authors:** Johanna Rienks, Janett Barbaresko, Ute Nöthlings

**Affiliations:** Department of Nutrition and Food Sciences, Nutritional Epidemiology, University of Bonn, Bonn 53115, Germany; j.barbaresko@uni-bonn.de (J.B.); noethlings@uni-bonn.de (U.N.)

**Keywords:** polyphenols, biomarkers, flavonoids, cardiovascular disease, mortality, observational, meta-analysis, enterolactone

## Abstract

Epidemiologic studies have suggested an inverse association between flavonoids and cardiovascular disease (CVD). However, the results might have been influenced by the use of dietary assessment methods, which are error prone. The aim of this paper was to systematically review and analyse the literature for evidence of associations between polyphenol biomarkers and CVD and mortality risk in observational studies. Eligible studies were identified through PubMed, Web of Science, and reference lists. Multivariable adjusted associations were extracted. Data were log-transformed and pooled using the random effects model. In total, eight studies were included, investigating 16 different polyphenol biomarkers in association with CVD and mortality. Blood and urine were used as biospecimens, and enterolactone, a lignan metabolite, was most often investigated. Three meta-analyses were conducted investigating the association between enterolactone, and all-cause and CVD mortality, and non-fatal myocardial infarction. A 30% and 45% reduced all-cause and CVD mortality risk were revealed at higher enterolactone concentrations. Furthermore, inverse associations were observed between polyphenol biomarkers and all-cause mortality, kaempferol, and acute coronary syndrome. There is evidence to suggest that enterolactone is associated with a lower CVD mortality risk. This emphasises the importance of the role of the microbiota in disease prevention. To strengthen the evidence, more studies are warranted.

## 1. Introduction

Cardiovascular diseases (CVD) are the leading cause of death worldwide [1]. By tackling modifiable lifestyle factors such as an unhealthy diet, most CVDs could in theory be prevented. A healthy diet containing plant-based foods [1] is abundant in bioactive compounds, such as polyphenols. Over 500 different heterogeneous molecular structures of polyphenols have been identified in plant foods [2]. Based on their structure, four groups of polyphenols can be distinguished, including flavonoids, phenolic acids, stilbenes, and lignans [3,4,5]. Of great interest to scientists is the group of flavonoids as their compounds are widely distributed in plant foods [6]. This group can be further classified into flavonols (main food sources: onions, curly kale, leeks, broccoli, apples, blueberries), flavanols (tea, grapes, cocoa), flavanones (citrus fruits), flavones (parsley, celery), anthocyanins (berries, black grapes), and isoflavones (soybeans) [3,7]. Also relatively abundant in plant foods are phenolic acids (coffee, outer part of fruits); however, with respect to disease risk, they have been investigated less often [5]. This is also the case for stilbenes, which are less dispersed in plant foods (wine, peanuts) [8]. Lignans, like flavonoids, have been investigated often and are found in linseed and cereals [5]. In the gut, lignans can be converted by microbiota to enterolactone (ENL) and enterodiol (END) [5], and can be detected in human biofluids.

The extensive research on polyphenols in animal and human studies has shown that these compounds possess a wide range of disease preventive properties including anti-inflammatory, antioxidant, and estrogenic activities [6]. However, because of the heterogeneity of findings across human studies, the role of polyphenols in CVD risk remains inconclusive. This might be due to the method used to assess the polyphenol intake. Most studies estimate polyphenol exposure of a participant’s diet from food composition tables such as the USDA database [9] and Phenol-Explorer [2]. However, these tables might be of limited use because only a very restricted number of foods have been analysed for their polyphenol content using different analytical techniques [3]. Furthermore, polyphenol values in foods fluctuate as a result of climate, soil, ripeness, processing, and storage [3]. To overcome these measurement errors and provide more accurate measures of polyphenol exposure, the use of biomarkers has been suggested [10]. In large epidemiologic studies, mostly single samples of serum, plasma, or urine are collected. Considering the relatively short half-life of most compounds, habitual exposure is probably best reflected in 24-h urine. Zamora-Ros et al. [11] showed that the total urinary polyphenol excretion from 24-h urine was correlated with dietary intake. Furthermore, creatinine normalised spot urine proved to be a suitable biomarker when adjusted for factors modifying creatinine excretion [11].

The aims of the current study were to: (1) systematically review the literature for evidence of associations between polyphenol biomarkers and all-cause mortality, CVD mortality, and CVD incidence in observational studies; and (2) conduct meta-analyses of individual biomarkers of polyphenols and outcomes where possible. Isoflavone biomarkers and chronic disease and mortality were covered elsewhere [12]. 

## 2. Methods 

This review was conducted according to the PRISMA guidelines [13] (Supplementary Table S1). A systematic search of the published literature was conducted in PubMed and Web of Science on 22 February 2017. The following search terms were used (both singular and plural): “biomarker”, “plasma”, “serum”, ”urine”, “urinary”, “excretion”, “concentration”, “level”, with “polyphenol”, “flavonoid”, “flavone”, “flavanone”, “flavonol”, “proanthocyanidin”, “anthocyanin”, “apigenin”, “luteolin”, “hesperetin”, “hesperedin”, “naringenin”, “kaempferol”, “quercetin”, “tamarixetin”, “matairesinol”, “epicatechin”, “epicatechin gallate”, “coumestrol”, “stilbene”, “resveratrol”, “tannin”, “lignans”, “enterolactone”, “enterodiol”, “enterolignan”, “pinoresinol”, “lariciresinol”, “secoisolariciresinol”, “matairesinol”, “phenolic acid”, “phytoestrogen”, with “cardiovascular disease”, “coronary heart disease”, “heart disease”, “CVD”, “heart disease”, “coronary artery disease”, “myocardial infarction”, “stroke”, “cerebrovascular disease”, “heart failure”, “mortality”, “death”, “cardiovascular mortality”, with “observational”, “epidemiologic”, “cohort”, “longitudinal”, “prospective”, “case-control”, “nested case-control”, not “animals” (using MeSH terms in PubMed).

### 2.1. In- and Exclusion Criteria

Two authors (JR and JB) independently screened the titles and abstracts of the publications. A third acted as a moderator (UN), to remove any discrepancies. Articles were retained for review if the following inclusion criteria were met: (1) investigation of multiple, adjusted associations between polyphenol biomarker(s) and CVD risk or mortality; (2) use of an observational study design; (3) the study involved humans; and (4) the study was published in a scientific journal (conference abstracts and comments were excluded). 

Articles were excluded if they met with at least one of the following criteria: (1) a dietary intervention was conducted prior to biospecimen sampling; or (2) only a dietary assessment method was used to assess polyphenol exposure. Finally, reference lists of all the included publications were screened to identify further articles meeting the inclusion criteria. No constraints were put on the language of the articles.

### 2.2. Data Extraction

From each article, the following details were extracted: first author, year of publication, country where the study was conducted, study design and cohort, characteristics of the participants (age, sex, number of cases/controls or cohort), follow-up time, specimen type, polyphenol biomarker(s), outcome(s), association measure, and confounding and matching variables. 

### 2.3. Quality Assessment

The quality of the included studies was assessed using the Newcastle-Ottawa scale developed for non-randomised studies [14]. The scale appraised three aspects including the selection of the study groups, the comparability of the groups, and the ascertainment of either outcome or exposure from cohort or nested case-control studies, respectively. The maximum number of ‘stars’ awarded to each section was four, two, and three for selection, comparability, and outcome/exposure, respectively. 

### 2.4. Statistical Analysis

Meta-analyses were conducted when at least two studies were available with a common exposure and outcome. This resulted in a total of three meta-analyses investigating the association between ENL, and all-cause and CVD mortality, and non-fatal myocardial infarction. For the meta-analyses, all effect sizes (HR, OR, RR) and 95% confidence intervals (CI) were log-transformed to maintain symmetry in the analysis. Standard errors were calculated from log CIs by subtracting the lower CI from the upper CI and subsequently dividing by 3.92. The *I*^2^ statistic was calculated to test the percentage of variation across studies due to heterogeneity [15]. Heterogeneity was assumed to be present because of differences in the study design and population. Therefore, we used a random-effects model that assumes a distribution of the true effect size by allowing both between- and within study variation [16]. Pooled estimates were visualized in forest plots. Potential publication bias was investigated by a visual inspection of the funnel plot, whereby asymmetry illustrated publication bias, and was tested quantitatively with the Egger’s test [17]. As CVD incidence and mortality differ between men and women, where possible, subgroup analyses were performed. The package ‘meta’ [18] in R [19] statistical software Version 3.3.2 was used to conduct the meta-analyses and ‘metabias’ was used to assess publication bias. A *p*-value < 0.05 was considered statistically significant.

## 3. Results

### 3.1. Search Results

**Figure 1:**
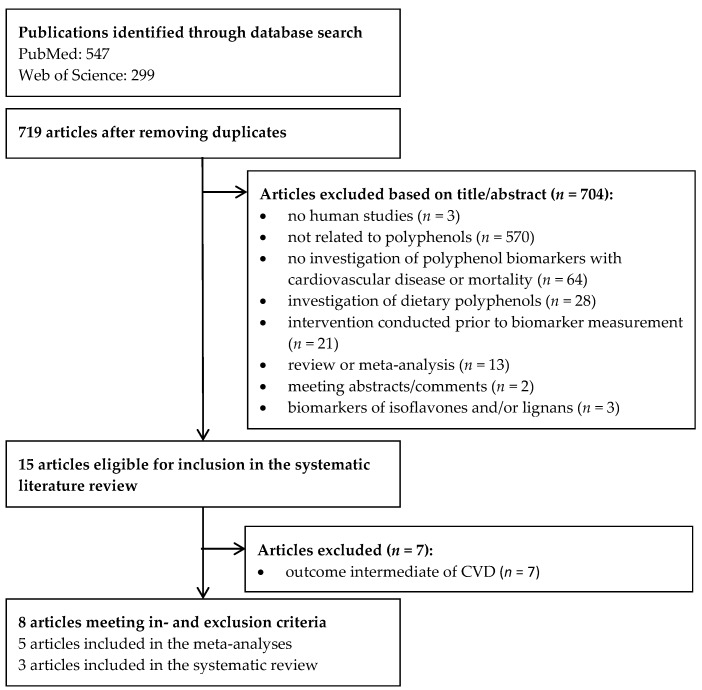
Flowchart of the study selection.

After removing duplicates, 719 studies remained (Figure 1). The titles and abstracts were screened. Ten full-texts were read and in total eight observational studies [20,21,22,23,24,25,26,27] were identified that investigated polyphenol biomarkers in association with mortality or CVD. In total, 16 polyphenolic compounds were investigated (Table 1). Five studies investigated ENL [21,22,23,24,25], two END [22,23], and one total polyphenols [26], resveratrol [27], lignans [23], flavonoids [20], flavonols [20], flavanones [20], flavones [20], and phloretin [20]. Two studies solely investigated all-cause mortality [26,27], two studied all-cause and CVD mortality [23,25], two studied CVD incidence and mortality [20,21], and three CVD incidence [21,22,24]. The characteristics and results of the included studies are presented in Table 2 and Table 3, respectively. The following biospecimens were used: two studies used 24 h urine [26,27], two used spot urine [20,23], three used serum [21,24,25], and one used plasma [22]. Six different study populations from five different countries were studied including Denmark, Finland, Italy, the Netherlands, and the USA. Four [23,25,26,27] studies had a prospective cohort study design, three [20,22,24] were nested case-control studies, and one [21] had a case-cohort design. All publications were of moderate to good quality with scores for both cohort and nested case-control studies ranging from five to eight stars (Supplementary Table S2). 

### 3.2. All-Cause Mortality

**Figure 2:**
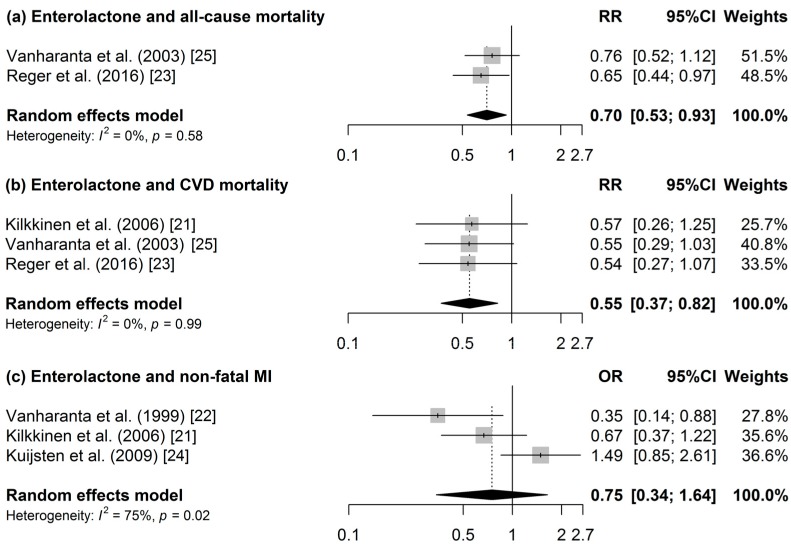
Forest of the association between enterolactone and (**a**) all-cause mortality; (**b**) CVD-mortality; and (**c**) non-fatal MI.

Four publications investigated all-cause mortality [23,25,26,27] (Table 3). The meta-analysis of two studies [23,25] revealed a 30% lower all-cause mortality risk at higher ENL concentrations (Figure 2A). Heterogeneity was not present and publication bias could not be indicated from the funnel plot. Furthermore, decreased mortality risks of 30%, 35%, and 35% were observed at higher total urinary polyphenol (TUP) [26], enterolignans [23], and ENL [23] concentrations, respectively. However, no associations were observed for resveratrol [27] and END [23].

### 3.3. CVD Incidence and Mortality

In total, six studies investigated CVD incidence [20,21,22,24] or mortality [21,23,25]. Pooling data for CVD mortality, a 45% reduced risk was revealed when the highest and the lowest quantile of ENL concentration were compared (Figure 2B). Heterogeneity and publication bias were not present. In a subgroup analysis of men, a similar result was found (RR (95% confidence interval (CI): 0.56 (0.34, 0.91)). When stratifying by sex, a 47% reduced non-fatal MI risk was observed (RR (95% CI): 0.53 (0.29, 0.98)). Inverse associations were also found between ENL and CHD mortality [25], and kaempferol excretion and acute coronary syndrome (ACS) [20]; however, no associations were observed for the other polyphenols in this study [20], and between END and CVD mortality in another study [23].

In the meta-analysis of three studies on ENL [21,22,24], no statistically significant association was identified for non-fatal MI. Heterogeneity was high (*I*^2^ = 75%) and there was no indication for publication bias according the Egger’s test. END was not statistically significantly associated with nonfatal MI [22]. 

## 4. Discussion

To our knowledge, this is the first study to give a complete overview of the published evidence of associations between polyphenol biomarkers and CVD risk and mortality in human population-based studies. Only eight studies were found, allowing meta-analyses with only two or three studies comparing ENL and mortality, CVD-mortality, or CHD incidence. The meta-analyses of all-cause and CVD mortality revealed a 30% and 45% reduced risk at higher ENL levels, respectively. No associations were observed in meta-analyses for incident MI and ENL concentrations. Indeed, the microbiota-derived lignan metabolites ENL and END were most frequently investigated. Single findings were observed between TUP and all-cause mortality, and kaempferol and ACS.

### 4.1. Comparison with other Studies on Dietary Polyphenols 

In line with the inverse association that was revealed in the present meta-analysis of all- cause mortality, is the 40% reduced risk observed for dietary lignans in Spanish community-dwelling elderly [28] and the 31% reduced risk for matairesinol found in elderly Dutch men [29]. No association was observed for total dietary lignans in this study [29]. In contrast to the reduced CVD mortality risk in the current meta-analysis, a prospective study from the Netherlands did not find an association with total dietary lignans; however, matairesinol again tended to be inversely associated [29]. 

No association was observed for total dietary polyphenols (TDP), as opposed to the statistically significant inverse association for TUP [26] in the same study. However, in a larger sample of a community-dwelling Spanish population, reduced risks of 37% and 52% were found for TDP and stilbenes (a group that includes resveratrol), respectively [28]. The result for stilbenes was not reflected in the resveratrol biomarker study [27] in the current review. 

All in all, there seems to be some evidence from biomarker studies linking microbiota-derived lignan biomarkers to mortality endpoints, but overall, only a few studies were available.

In agreement with the null-finding for non-fatal MI, are the results from the EPIC-Prospect study [30]. The statistically significant reduced ACS risk of the kaempferol biomarker [20] is in line with the results of a meta-analysis [31] and a prospective Italian study [32]. The null associations found for flavonoid biomarkers and ACS [20] are consistent with a meta-analysis of total dietary flavonoids and their subgroups [31], dietary flavonols and flavanones, in two prospective studies [33,34], and flavones in three prospective studies [32,33,34]. In contrast, a statistically significant decreased CVD risk trend was observed for dietary dihydrochalcone intake [34] (class of phloretin [20]). 

Evidence from biomarker studies is scarce and more studies on all compounds are warranted. 

### 4.2. Validity of the Biomarker Measure

The main advantage of using biomarkers for an exposure assessment over dietary assessment methods is that these account for inter-individual differences in absorption, distribution, and metabolism [3]. Nevertheless, biomarker concentrations are influenced by several factors, of which some are known and others are yet to be determined. Treatment with antibiotics is a known factor that influences the formation of lignan metabolites [35]. The failure to account for antibiotic use is an important limitation of the studies included in the meta-analyses [21,22,23,24,25]. The number of antibiotic treatments and the time since the last treatment affect the microflora and were shown to result in lower ENL concentrations, even 12–16 months after treatment [35]. Since all of the studies included in the meta-analysis did not consider antibiotic use, their observed risks are likely to be attenuated. The included studies used single samples of blood and urine, which were predominantly sampled in a fasting state. This raises the question of which biomarker is better correlated with biological relevance, especially considering the potential differences between individuals. The absorption, peak plasma, metabolism, and excretion of polyphenols are likely to be determined by individual physiology. Therefore, a single sample may not provide a reliable marker for the total bioavailable concentration throughout time. Considering the relatively short half-lives of most polyphenolic compounds, 24 h urine should contain all of the ingested compounds and/or their metabolites and conjungates.

### 4.3. Strengths and Limitations of the Present Review

The main strength of the present study is the inclusion of several meta-analyses of polyphenol biomarkers. To our knowledge, these are the first meta-analyses of lignan biomarkers representing an internal dose. Although we only conducted meta-analyses on two (for all-cause mortality) and three (for CVD mortality and non-fatal MI) studies, a good overview of the level of consistency across the studies is provided. 

All of the studies included in the meta-analysis of CVD-mortality used Cox proportional hazard models, either reporting HR or RR, which have the same meaning and are used interchangeably. Because the highest and lowest exposure quantiles were compared, a dose-response relationship could not be derived. Furthermore, the remaining three studies included in the systematic literature were slightly heterogeneous with regard to the polyphenol biomarker measured. However, this only emphasizes the complexity of the polyphenol exposure and the need to investigate the role of the individual polyphenols in cardiovascular disease and mortality. Another strength is that the quality of the included studies was judged to be moderate to good. Beside antibiotic use, another potential confounder, creatintine excretion, was only considered in the study by Reger et al. [23]. The failure to account for urinary creatinine excretion could result in an over- or underestimation of polyphenol concentrations, depending on the dilution of the urine. From the visual inspection of the funnel plots and interpretation of the Egger’s test, publication bias was not present. However, significant heterogeneity was observed in the meta-analysis of MI. This was further explored in the sensitivity analysis. By excluding one study at a time, we observed that the study by Kuijsten et al. (2009) introduced the heterogeneity, as after exclusion, we found an *I*^2^ of 25.6%. The heterogeneity could have resulted from sex differences, as the inclusion of men did result in an inverse association. Another explanation might be the difference in the biospecimens or the lower ENL concentrations in this study [22], where the cut-off in the highest quartile was >17.5 nmol/L. In comparison, ENL concentrations were much higher in the studies by Vanharanta et al. (1999) [24] and Kilkkinen et al. (2006) [21], namely >30.1 and >28.24 nmol/L, respectively. This could be simply explained by differences in dietary habits between the Netherlands and Finland, or because of the differences in biomarkers used to measure ENL. Furthermore, in addition to the search in two large databases and the articles’ reference lists, a search in gray literature could have resulted in the identification of unpublished papers. Language bias was prevented by imposing no restrictions on language; in spite of this, no non-English written publications were retrieved that met the inclusion criteria. Interestingly, only one study [26] investigated polyphenol biomarkers and dietary polyphenols in the same study population. Therefore, the emerging question is whether these approaches would provide stronger results when biomarkers are measured in conjunction with dietary intake. The current review, however, suggests that the polyphenol biomarkers strongly reflect internal doses, which are not necessarily strongly associated with long-term intake due to the many factors influencing bioavailability [3,36].

## 5. Conclusions

A number of studies have been published reporting on the associations between polyphenol biomarkers, and all-cause, CVD mortality, and CVD risk. In the meta-analyses, inverse associations were revealed between ENL and all-cause and CVD mortality. Furthermore, in the systematic review, inverse associations were observed for TUP with all-cause mortality and ACS with kaempferol. For future research, comparability across studies should be improved to enable a quantitative analysis. Furthermore, it is recommended that groups investigate individual polyphenolic compounds and even metabolites instead of total polyphenols from different groups and classes. It might be worth considering collecting multiple biospecimen samples or 24 h urine samples that reflect circadian polyphenol exposure, although this might not be desirable in large cohort studies as it places a burden on the study participants.

## Figures and Tables

**Table 1:** Frequency of studies reporting on polyphenol biomarkers in association with mortality and cardiovascular disease (CVD).

Polyphenolic Group Compound	Reference	Frequency of Investigation	Mortality	CVD Mortality	CVD Incidence
Total polyphenols	[26]	1	1		
Total flavonoids	[20]	1			1
Total flavonols	[20]	1			1
• Kaempferol		1			1
• Quercetin		1			1
• Tamarixetin		1			1
• Isorhamnetin		1			1
Total flavanone	[20]	1			1
• Naringenin		1			1
• Hesperetin		1			1
Total flavone	[20]				
• Apigenin		1			1
Phloretin	[20]	1			1
Resveratrol	[27]	1	1		
Lignans	[23]	1	1	1	
• Enterolactone	[21,22,23,24,25]	5	2	3	3
• Enterodiol	[22,23]	2	1	1	1

**Table 2:** Characteristics of studies included in this systematic literature review investigating the association between polyphenol biomarkers and cardiovascular disease and mortality.

Author (Year) Country	Design	Study Name	Specimen	Biomarker	Cases, *n*	Cohort, *n* (Sex % Women)	Age, Year	Follow-Up, *y* ^1^	Outcome
Zamora-Ros et al. (2013) Italy [26]	ps	InCHIANTI	24 h urine	POLY	274	807 (58.7)	≥65	12	All-cause mortality
Semba et al. (2014) Italy [27]	ps	InCHIANTI	24 h urine	RES	268	783 (41.4)	≥65	9	All-cause mortality
Reger et al. (2016) USA [23]	ps	NHANES	Spot urine	ELIG, ENL, END	108	5179 (52.4)	≥18	5	CVD mortality
					290				All-cause mortality
Vanharanta et al. (2003) Finland [25]	ps	KIHD	Serum	ENL	70	1889 (0)	42–60	12.2	CHD mortality
					103				CVD mortality
					242				All-cause mortality
Kilkkinen et al. (2006) Finland [21]	caco	ATBC	Serum	ENL	340	760 (0)	50–69	11.1	All CHD events
					205				Nonfatal MI
					135				Coronary death
Bredsdorff et al. (2013) Denmark [20]	ncc	DCH	Afternoon spot urine	flavonoids, phloretin, FLAVO, ISO, KAE, QUE, TAM, FLAVAN, HES, NAR, API	393	786 (20.1)	50–64	8 (TP)	Acute coronary syndrome
Kuijsten et al. (2009) The Netherlands [22]	ncc	Monitoring Project on CVD risk factors	Plasma	ENL, END	236	519 (31.1)	20–59	4.5	Nonfatal MI
Vanharanta et al. (1999) Finland [24]	ncc	KIHD	Serum	ENL	167	334 (0)	42–60	10	Acute coronary events

^1^ Mean or median (med) follow-up time, total study period (TP) was calculated, when follow-up time was not reported, by subtracting the year of last follow-up from the year of specimen collection. API, apigenin; ATBC, Alpha-Tocopherol, Beta-Carotene Cancer Prevention study; caco, case-cohort; CHD, coronary heart disease; CVD, cardiovascular disease; DCH, Diet Cancer and Health study; END, enterodiol; ENL, enterolactone; ELIG, enterolignan; FLAVAN, flavanones; FLAVO, flavonol; HES, hesperetin; InCHIANTI, Invecchiare in Chianti; ISO, isorhamnetin; KAE, kaempferol; KIHD, Kuopio Ischaemic Heart Disease Risk Factor Study MI, myocardial infarction; *n*, number; NAR, naringenin; ncc, nested case-control study; NHANES, National Health and Nutrition Examination Survey; POLY, polyphenols; ps, prospective study; QUE, quercetin; RES, resveratrol; TAM, tamarixetin; TP, total period.

**Table 3:** Results of the studies included in this systematic literature review of studies investigating the biomarkers of polyphenols with mortality and cardiovascular disease.

Author Year, Country	Biomarker	Endpoint	Association (95% CI) of Extreme Quantiles		P-trend	Confounders (C) and Matching (M) Variables
**All-cause mortality**						
Zamora-Ros et al. (2013) Italy [26]	POLY	All-cause mortality	HR Q3/Q1	0.70 (0.49, 0.99)	0.05	C: age, sex, education, BMI, alcohol intake, smoking status, renal function, PA, CVD, DM, cancer, COPD, dementia, Parkinson’s disease, energy intake only for TDPs
Semba et al. (2014) Italy [27]	RES	All-cause mortality	HR Q4/Q1	0.80 (0.54, 1.17)	0.43	C: age, sex, education, BMI, PA, total cholesterol, HDL, MMSE score, mean arterial BP, and chronic diseases: CHD, stroke, heart failure, cancer, DM, peripheral artery disease, chronic kidney disease
Reger et al. (2016) USA [23]	ELIG	All-cause mortality	HR Q3/Q1	0.65 (0.43, 0.96)	0.019	C: age, education, smoking status, BMI, total energy intake, sodium intake, urinary creatinine
	ENL			0.65 (0.44, 0.97)	0.014	
	END			0.98 (0.67, 1.43)	0.85	
Vanharanta et al. (2003) Finland [25]	ENL	All-cause mortality	HR Q4/Q1	0.76 (0.52, 1.12)	0.09	C: age, year of examination, year of serum ENL measurement, DM, hypertension, urinary excretion of nicotine metabolites, BMI, alcohol, LDL, HDL, dietary intake of fiber, folate, vitamins C and E, saturated fatty acids
**CVD incidence and mortality**						
Bredsdorff et al. (2013) Denmark [20]	flavonoids	Acute coronary syndrome	OR Q5/Q1	0.63 (0.37, 1.05)	0.32	C: period of analysis, BMI, waist circumference, smoking, hypertension, DM, alcohol, hypercholesterolemia, PA, level of school education M: sex, age, smoking, time specimen collection
	phloretin			0.87 (0.54, 1.39)	0.46	
	FLAVO			0.83 (0.50, 1.36)	0.46	
	ISO			0.72 (0.41, 1.25)	0.15	
	KAE			0.55 (0.32, 0.92)	0.12	
	QUE			0.94 (0.58, 1.51)	0.65	
	TAM			1.06 (0.65, 1.74)	0.78	
	FLAVAN			0.68 (0.41, 1.12)	0.26	
	HES			0.72 (0.43, 1.18)	0.34	
	NAR			0.63 (0.38, 1.02)	0.12	
	API			1.20 (0.73, 1.96)	0.73	
Kuijsten et al. (2009) the Netherlands [22]	ENL	Nonfatal MI	OR Q4/Q1	1.49 (0.85, 2.61)	0.140	C: current smoking, BMI, systolic blood pressure, total cholesterol, HDL cholesterol, ratio total/HDL cholesterol, current smoking, SBP, ratio total/HDL cholesterol
	END			1.18 (0.67, 2.07)	0.860	M: age (5 years), sex, study center
Vanharanta et al. (1999) Finland [24]	ENL	Acute coronary events	OR Q4/Q1	0.35 (0.14, 0.88)	0.01	C: serum apolipoprotein B, dietary iron intake, fam hist of CHD, ischaemic findings on exercise test, dietary calcium intake, urinary excretion of nicotine metabolites, DM, SBP, maximum oxygen uptake
						M: age, examination year, place of residence
Kilkkinen et al. (2006) Finland [21]	ENL	All CHD events	RR Q5/Q1	0.63 (0.33, 1.11)	0.07	C: age, BMI, total and HDL cholesterol, DBP, SBP, alcohol intake, nr of smoking years and cigarettes smoked per day, hist of CHD and DM, fasting time, dietary factors
		Nonfatal MI		0.67 (0.37, 1.23)	0.10	
		Coronary death		0.57 (0.26, 1.25)	0.18	
Reger et al. (2016) USA [23]	ELIG	CVD mortality	HR Q3/Q1	0.48 (0.24, 0.97)	0.07	C: age, education, smoking status, BMI, total energy intake, sodium intake, urinary creatinine
	ENL			0.54 (0.27, 1.07)	0.10	
	END			0.71 (0.87, 1.78)	0.52	
Vanharanta et al. (2003) Finland [25]	ENL	CHD mortality	HR Q4/Q1	0.44 (0.20, 0.96)	0.03	C: age, year of examination, year of serum ENL measurement, DM, hypertension, urinary excretion of nicotine metabolites, BMI, alcohol, LDL, HDL, dietary intake of fiber, folate, vitamins C and E, saturated fatty acids
		CVD mortality		0.55 (0.29, 1.01)	0.04	

API, apigenin; BMI, body mass index; CHD, coronary heart disease; COPD, chronic obstructive pulmonary disease; CVD, cardiovascular disease; DBP, diastolic blood pressure; DM, diabetes mellitus; ELIG, enterolignan; END, enterodiol; ENL, enterolactone; fam hist, family history; FLAVA, flavanol; FLAVAN, flavanones; FLAVO, flavonol; ISO, isorhamnetin; HDL, high-density lipoprotein; HES, hesperetin; hist, history; HR, hazard ratio; ISO, isorhamnetin; KAE, kaempferol; LDL, low density lipoprotein; MMSE, mini-mental state examination; NAR, naringenin; OR, odds ratio; PA, physical activity; POLY, polyphenols; Q, quantile; QUE, quercetin; RES, resveratrol; RR, rate ratio; SBP, systolic blood pressure; TAM, tamarixetin; TDPs, total dietary polyphenols.

## Supplementary Materials

The following are available online at www.mdpi.com/2072-6643/9/4/415/s1, Table S1: PRISMA checklist, Table S2: Results of quality assessment of included studies using the Newcastle-Ottawa scale for non-randomised studies.
